# Cadmium, Lead, and Other Metals in Relation to Semen Quality: Human Evidence for Molybdenum as a Male Reproductive Toxicant

**DOI:** 10.1289/ehp.11490

**Published:** 2008-07-01

**Authors:** John D. Meeker, Mary G. Rossano, Bridget Protas, Michael P. Diamond, Elizabeth Puscheck, Douglas Daly, Nigel Paneth, Julia J. Wirth

**Affiliations:** 1 Department of Environmental Health Sciences, University of Michigan School of Public Health, Ann Arbor, Michigan, USA; 2 Department of Animal and Food Sciences, University of Kentucky, Lexington, Kentucky, USA; 3 Department of Epidemiology, Michigan State University, East Lansing, Michigan, USA; 4 Department of Obstetrics and Gynecology, Wayne State University, Detroit, Michigan, USA; 5 Grand Rapids Fertility and IVF, Grand Rapids, Michigan, USA

**Keywords:** biomarkers, epidemiology, exposure, fertility, metals, sperm

## Abstract

**Background:**

Evidence on human semen quality as it relates to exposure to various metals, both essential (e.g., zinc, copper) and nonessential (e.g., cadmium, lead), is inconsistent. Most studies to date used small sample sizes and were unable to account for important covariates.

**Objectives:**

Our goal in this study was to assess relationships between exposure to multiple metals at environmental levels and human semen-quality parameters.

**Methods:**

We measured semen quality and metals in blood (arsenic, Cd, chromium, Cu, Pb, manganese, mercury, molybdenum, selenium, and Zn) among 219 men recruited through two infertility clinics. We used multiple statistical approaches to assess relationships between metals and semen quality while accounting for important covariates and various metals.

**Results:**

Among a number of notable findings, the associations involving Mo were the most consistent over the various statistical approaches. We found dose-dependent trends between Mo and declined sperm concentration and normal morphology, even when considering potential confounders and other metals. For example, adjusted odds ratios (ORs) for below-reference semen-quality parameters in the low, medium, and high Mo groups were 1.0 (reference), 1.4 [95% confidence interval (CI), 0.5–3.7], and 3.5 (95% CI, 1.1–11) for sperm concentration and 1.0 (reference), 0.8 (95% CI, 0.3–1.9), and 2.6 (95% CI, 1.0–7.0) for morphology. We also found preliminary evidence for interactions between Mo and low Cu or Zn. In stratified analyses, the adjusted ORs in the high Mo/low Cu group were 14.4 (1.6, 132) and 13.7 (1.6, 114) for below-reference sperm concentration and morphology, respectively.

**Conclusions:**

Our findings represent the first human evidence for an inverse association between Mo and semen quality. These relationships are consistent with animal data, but additional human and mechanistic studies are needed.

The general population is exposed to metals at low concentrations either voluntarily through supplementation or involuntarily through intake of contaminated food and water or contact with contaminated soil, dust, or air. Some metals, such as cadmium, lead, arsenic, and mercury, are nonessential xenobiotics that can be measured in most of the general population [Centers for Disease Control and Prevention (CDC) 2005]. Because widespread human exposure and body burden have been demonstrated, there is growing concern for adverse health effects associated with low-level exposures encountered in the environment. Human and animal evidence suggests that these metals may have adverse impacts on male reproductive health at relatively low levels. For example, Cd has been linked to poor human semen quality and DNA damage (Telisman et al. 2000; Xu et al. 2003); Pb may adversely affect sperm shape, motility, and DNA integrity (Eibensteiner et al. 2005; Hernandez-Ochoa et al. 2005; Jurasovic et al. 2004; Telisman et al. 2007); and methyl-mercury is associated with sperm abnormalities in subfertile males (Choy et al. 2002). However, human data on nonoccupational exposure to these metals has been limited (e.g., Hg), lacking (e.g., As), or inconsistent across studies (e.g., Cd ). We designed the present study to explore relationships between these nonessential metals and semen quality among men with exposure levels that are likely to be representative of those found among the U.S. general population.

Several other metals, such as chromium, copper, manganese, molybdenum, selenium, and zinc, are essential for good health but may be harmful above certain levels [Agency for Toxic Substances and Disease Registry (ATSDR) 2003, 2004, 2005; Greger 1999; Institute of Medicine (IOM) 2001]. For example, Cr, Mn, and Cu, which act as cofactors for a variety of important enzymes, have been associated with reduced semen quality in rodents and in humans (Adejuwon et al. 1996; Huang et al. 2000; Kumar et al. 2005; Telisman et al. 2000; Wirth et al. 2007). Mo is also an important cofactor for a limited number of human enzymes and has demonstrated reproductive toxicity in animal studies (IOM 2001). On the other hand, low doses of metals such as Cu, Se, and Zn may have protective effects on male reproductive outcomes (Benoff et al. 1997; Evenson et al. 1993; Lyubimov et al. 2004; Olson et al. 2005) and may assist in counteracting the effects of Cd, Pb, or other metals (Telisman et al. 2000; Xu et al. 2003). Because the potential exists for a number of metals to positively or negatively affect male reproduction either individually or together, we also included these metals in our analysis. This work represents the most comprehensive study to date on metal exposures at environmental levels and human semen quality.

## Materials and Methods

### Subject recruitment

We recruited participants at two Michigan infertility clinics without knowledge of male factor infertility as part of an ongoing epidemiologic study of environmental contaminants and male reproduction. Because couples may present at the clinics for problems relating to either male or female fertility problems (or both), the study population includes fertile men and men with a range of fertility problems. Men between 18 and 55 years of age currently attempting to conceive a pregnancy with their partner were enrolled. We excluded men with diabetes, thyroid or adrenal disorders, genetic disorders related to fertility, testicular cancer, bilateral orchiectomy, or taking hormone therapy. The protocols of the study were approved by the committees on research ethics at all participating institutions, and informed consent was obtained from all participants.

### Semen parameters

Semen samples were collected using standard protocols, and semen analysis was conducted by andrology technicians following the World Health Organization protocol (WHO 1999). The semen parameters we investigated in this study were semen volume, sperm count, sperm concentration (million sperm per milliliter), percent motile sperm, and sperm morphology. The concentration of immobilized sperm was determined using a counting chamber. Sperm motility was evaluated at 1 hr after collection. Percent motile was the sum of the percentages with rapid linear progression (3 to ≥4) and slow linear progression (≥2). Sperm morphology (percent normal forms) was determined using air-dried smears stained with a modified Wright-Giemsa stain. At least 100 sperm in four different areas of the slide were evaluated according to Kruger’s strict criteria (Kruger et al. 1988).

### Measurement of metals

Whole venous blood was collected using stainless-steel needles into 2-mL plastic tubes containing EDTA (prescreened for Hg, Cd, and Pb) and stored at −20°C. Samples were assayed for As, Cd, Cr, Cu, Pb, Mn, total Hg, Mo, Se, thallium (Tl), and Zn. Controls for all metals included human or bovine blood spiked with known quantities of each metal. In addition, we analyzed Cd, Pb, and Hg with regard to standardized human reference blood containing known quantities of each metal. We analyzed all samples using an Elan DRC plus ion-coupled plasma mass spectrometer (PerkinElmer, Waltham, MA). The limits of detection (LOD) for the blood metal levels were as follows: As, 4.0 μg/L; Cd, 0.2 μg/L; Cr, 0.5 μg/L; Pb, 0.3 μg/dL; Mn, 1.0 μg/L; total Hg, 0.2 μg/L; Mo, 1.0 μg/L; Se, 5 μg/L; Tl, 0.1 μg/L; and Zn, 50 μg/L.

### Statistical analysis

We performed data analysis using SAS, version 9.1 (SAS Institute Inc., Cary, NC). We calculated descriptive statistics on subject demographics, along with the distributions of blood metal concentrations and semen-quality parameters. Bivariate analysis was conducted between all semen quality, metal concentration, and variables to investigate differences between distributions or categories and the potential for confounding. Correlations or differences between groups were tested statistically using parametric or nonparametric methods where appropriate.

Because of the high proportion of samples with values < LOD for a number of the metals, we categorized these variables into groups. At least three groups were formed for each metal to investigate dose-dependent relationships. Metals with > 75% of samples > LOD were categorized into quartiles. For the other metals, grouping cut-points were determined by the percentage of samples > LOD. The low group consisted of values < LOD for each metabolite, whereas the medium and high groups consisted of equal-sized bins among the detected values.

We assessed the association between exposure categories for each metal and sperm concentration, motility, and morphology by multiple logistic regression, where subjects were dichotomized as either greater than or less than WHO (1999) reference levels for sperm count (40 million total sperm), concentration (20 million sperm/mL), and motility (50% motile sperm). We used the strict criteria (4% normal) as a cutoff for sperm morphology (Kruger et al. 1988). Reference subjects had values greater than the reference level for all three parameters. Age, body mass index (BMI), race, abstinence period, and smoking were considered as covariates. A substantial proportion of men (27%) chose not to provide information on abstinence period. Although abstinence period may be positively associated with sperm concentration and inversely associated with sperm morphology (Meeker et al. 2007), it is unlikely to be associated with metal concentrations in blood. Thus, because abstinence period was not likely to act as a confounder in these data, we did not include it in the primary analyses in an effort to improve statistical power.

We also used multiple linear regression to assess associations between metal categories and continuous measures of semen quality. Sperm count and concentration were transformed using the natural logarithm, whereas all other semen-quality measures (motility, morphology, and semen volume) were modeled untransformed. We considered statistical and biologic factors in selecting which covariates to include in adjusted models. Because it may be appropriate to consider simultaneously the impact of multiple metals on semen quality (Jurasovic et al. 2004; Telisman et al. 2007), we also constructed full logistic and linear regression models for each semen-quality parameter when considering all metals and covariates together. We used the backward elimination procedure and set alpha at 0.10 for variables to be retained in the model. We then added covariates not retained in the final model individually to further explore evidence of confounding (i.e., causing > 10% change in effect estimates for metals in the final model). Mn and Se were excluded in the analysis of associations between individual metals and semen quality because they have been previously reported (Wirth et al. 2007), but they were included for consideration in the full models.

Finally, we explored evidence of interaction between metals. Because we lacked the sample size and statistical power to include interaction terms in the models, we performed this exploratory analysis by qualitatively comparing effect estimates from statistical analyses on a metal of concern (e.g., Cd or Pb) while stratifying on a metal that may be protective against metal-related adverse effects on semen quality (e.g., Zn, Cu, or Se).

## Results

We recruited a total of 219 men into the study who provided information on semen quality and metal concentrations in blood. Sperm morphology data were not available for 31 men because critical morphology was not performed for some couples who, for a number of possible reasons or circumstances, were destined for *in vitro* fertilization treatment. For all statistical analyses conducted, the results for total sperm count in relation to metals were similar to those for sperm concentration, so they are not presented here. Demographic data stratified by semen-quality reference-value categorizations are presented in Table 1. Participants were mostly white (76%), with a median age of 34 years. There were some differences in demographic categories among the semen-quality groups, such as percentage of current smokers in the below-reference group for sperm concentration and motility, but none of the differences were statistically significant (*p* > 0.05). Distributions of metal concentrations measured in blood and semen-quality parameters are presented in Table 2. As, Cu, Pb, total Hg, and Zn were > LOD for most samples. Fifty percent of samples had Cd and thallium levels > LOD, whereas 30% of samples had levels > LOD for Cr and Mo.

We conducted preliminary comparisons between variables using parametric or non-parametric correlations or hypothesis tests, depending on the detection rate and distribution of the individual variables (all reported associations had *p* < 0.05). Age was inversely associated with sperm motility and positively associated with blood Pb and Hg concentrations. Current smoking was inversely associated with both semen volume and sperm motility. Current smoking was also associated with increased Cd and Cu concentrations in blood and with decreased Zn. BMI was positively associated with Cu but not with any of the semen-quality parameters. Nonwhite race was positively associated with Cd, Cu, and Pb but inversely associated with Se and semen volume. Finally, income was inversely associated with Cd, Cu, and Pb but positively associated with Hg and semen volume.

Results from crude logistic models (data not shown) were similar to those adjusted for age and current smoking (Table 3). We chose to include age and current smoking in the models because they were the only variables associated with both metal concentrations and semen quality (with the exception of semen volume). The main difference from the crude models was a suggestive dose-dependent trend for increased odds of below-reference motility among increasing Pb quartiles (*p* for trend = 0.07) that became weaker after adjustment for age and smoking (*p* for trend = 0.18). In the adjusted models, As levels > 25th percentile were associated with increased odds of below-reference semen parameters. However, we observed the highest odds ratios (ORs) for the second As quartile, and the dose response was not monotonic among groups with increasing As levels. The second quartile for Cu was also associated with some significantly increased ORs compared with the lowest Cu quartile for sperm concentration and motility, although there was a lack of trend. We found a significant trend for increased ORs for below-reference sperm morphology with increasing Cu quartiles. For Mo groups, there was a statistically significant trend for increased odds of below-reference sperm concentration and a suggestive trend for increased odds of below-reference sperm morphology. Compared with those with Mo levels < LOD, the highest Mo group (> 85th percentile) was associated with 3.5-fold [95% confidence interval (CI), 1.1–10.8] increased odds of having below-reference sperm concentration and 2.6-fold (95% CI, 1.0–7.0) increased odds of having below-reference sperm morphology.

Cd was inversely associated with semen volume in crude linear regression models (data not shown), but this association did not persist after adjusting for age and current smoking (Table 4). Cu was associated with significant decreasing trends for both semen volume and normal sperm morphology; men in the highest Cu quartile had, on average, 0.6 mL less semen volume and a 2% decline in sperm with normal morphology compared with men in the lowest Cu quartile. Zn was also associated with a suggestive decline in semen volume. In these adjusted linear regression models, Mo groups were associated with suggestive decreasing trends in sperm concentration and morphology (Table 4), which was consistent with the trends for increased ORs for below-reference semen-quality parameters in the multiple logistic regression (Table 3). In addition, the high-Mo group was associated with a suggestive 5.4% decline in sperm motility (95% CI, −11.2 to 0.4; *p* = 0.06).

Because it may be appropriate to consider the impact of multiple metals on semen quality simultaneously (Jurasovic et al. 2004; Telisman et al. 2007), we considered all metals and covariates together in logistic and linear regression models for each semen parameter. In the final logistic regression models, Mo remained a predictor of both sperm concentration and morphology (Table 5). When adjusting for other metals and covariates, the high-Mo group was associated with an OR of 6.3 (95% CI, 1.6–25.0) for below-reference sperm concentration and an OR of 3.4 (95% CI, 1.2–9.7) for below-reference sperm morphology. Pb and Mn were also associated with increased odds of below-reference sperm concentration, whereas Cd and Hg were associated with decreased ORs. In multiple linear regression, we retained none of the metals in the sperm concentration model (data not shown). However, in accordance with the other modeling strategies, Mo was inversely associated with sperm morphology (Table 6), as was Cu. As and Zn were positively and inversely associated with semen volume, respectively.

We explored evidence for interaction by repeating logistic and linear models (adjusted for age and current smoking) for Cd, Pb, and Mo while stratifying on potentially protective metals. We found no evidence for interactions involving Cd or Pb with Zn, Cu, or Se in logistic or linear models (data not shown). For Mo, we found evidence for interaction in the associations with sperm concentration and morphology when stratifying by Cu and Zn. ORs for below-reference sperm concentration and morphology for the high Mo group were 14.4 (95% CI, 1.6–132.0) and 13.7 (95% CI, 1.6–114.0), respectively, when we limited analysis to subjects with blood Cu concentrations less than the median. Likewise, these ORs were 16.0 (95% CI, 1.6–161.0) and 10.4 (95% CI, 1.2–87.8), respectively, when limiting analysis to subjects with blood Zn concentrations less than the median. We observed similar evidence in linear regression models for Mo stratified by Cu or Zn. Adjusting for age and current smoking, the high-Mo group was associated with a 0.72 (95% CI, −1.3 to −0.14) decline in natural-log–transformed sperm concentration and a 2.7% (95% CI, −4.9 to −0.46) decline in sperm morphology when we limited the analyses to men with Cu levels less than the median. These estimates were stronger than those for the high-Mo among all men presented in Table 3 [regression coefficients were −0.29 (95% CI, −0.72 to 0.13) for sperm concentration and −1.39 (95% CI, −2.01 to 0.52) for sperm morphology]. Results were similar when stratifying by Zn, although the statistically significant effect estimates for the high-Mo group were not quite as large (i.e., they were closer to zero).

## Discussion

Among a number of notable findings involving the relationship between several metals and semen quality, some of which may be chance findings because of the number of comparisons that were made, the associations involving Mo appeared to be the most consistent across the various statistical approaches we used. We found significant or suggestive associations and dose-dependent trends between Mo in blood and declined sperm concentration and morphology, even when considering numerous covariates and blood concentrations of other metals. In addition, we found qualitative evidence for an interaction between Mo and low Cu or Zn levels.

Mo is a ubiquitous trace element found in food and drinking water and is present in multivitamin/multimineral supplements. Among foods, Mo is found at higher concentrations in leafy vegetables and legumes (Vyskocil and Viau 1999). Mo concentrations in food, especially plants, depend greatly on species and soil characteristics. Concentrations of Mo in drinking water also likely vary. It was present at detectable levels in most (62%) groundwater or surface water samples tested in the United States over the last 15 years, collected mostly during land use surveys, with concentrations ranging from < 0.05 μg/L to more than 4,500 μg/L (U.S. Geological Survey 2008). Human exposure may be elevated in areas involved in the mining of Mo ore, or can also result from certain industrial operations (CDC 2005). Mo is used in the manufacture of electronic parts, glass, ceramics, and lubricants; in the production of catalysts and pigments; in steel alloys; and in chemical reagents found in hospital laboratories (CDC 2005; Pandey and Singh 2002).

To our knowledge, this is the first study to investigate the association between Mo and human semen quality. However, the reproductive toxicity of Mo has been described in several animal studies dating back as far as 1954, where male rats demonstrated decreased fertility when exposed to high levels of Mo for 13 weeks (IOM 2001; Jeter and Davis 1954; Vyskocil and Viau 1999). In a recent study of catfish from polluted waters in the Vietnamese Mekong Delta area, Yamaguchi et al. (2007) found significant inverse associations between tissue Mo, Pb, and As concentrations and gonadosomatic index (gonad weight/body weight × 100). Pandey and Singh (2002) reported a degeneration of testicular morphology and function and dose-dependent declines in sperm concentration, motility, and normal morphology in a study of rats after oral administration of sodium molybdate. The authors also reported evidence of male-mediated embryotoxicity (e.g., reduced implantation, increased pre- and postimplantation losses, and reduced fetal growth) associated with sodium molybdate exposure.

Animal studies also suggest the interaction of Mo with other minerals, namely, Cu. Mo has a chelating effect on Cu and has been associated with impaired Cu use in animal studies (Lyubimov et al. 2004); also, individuals who are deficient in Cu intake or have a Cu metabolism dysfunction may be at increased risk for Mo toxicity (IOM 2001). In a study of rams, Van Niekerk and Van Niekerk (1989) found lower semen volume and sperm concentration, motility, and normal morphology in a Cu-deficient group, created through supplementation with Mo and sulfate, compared with a control group that was given additional Cu supplementation. The semen-quality measures returned to normal when the Cu deficiency was reversed. A more recent study in rats found that tetrathiomolybdenum caused a reduction in epididymal weights, sperm concentration, motility, and normal morphology at high dose levels (Lyubimov et al. 2004). Interestingly, dietary Cu supplementation prevented the adverse effects on sperm at the same high Mo dose levels in the study (Lyubimov et al. 2004). These studies are consistent with our qualitative evidence for an interaction between Mo and low Cu in exploratory analyses, where we found effect estimates for Mo that were considerably higher among men with lower Cu (or Zn) concentrations in blood. Although Cu supplementation has been shown to be effective in protecting against adverse effects on semen quality in animal studies (Lyubimov et al. 2004; Van Niekerk and Van Niekerk 1989), caution should be taken when extrapolating this effect to humans. Because we also found evidence of an inverse association between high Cu levels and semen quality, which is consistent with a number of animal and human studies (Battersby et al. 1982; Huang et al. 2000; Massanyi et al. 2004; Skandhan 1992; Yuyan et al. 2007), more research is needed before advising Cu supplementation in men with high blood levels of Mo.

The results of the present study are consistent with our previous findings among an overlapping population of men. In an earlier study, we found that Mn was negatively associated with sperm concentration (Wirth et al. 2007), a relationship that we also observed in the present study when concurrently considering other metals (Table 5). Additionally, in a recent statistical methods development investigation, undertaken to address the issue of simultaneous exposures to multiple metals using μ statistics (Ramamoorthi et al. 2008), Cu and Mo as a pair were negatively correlated with total motile sperm and with normal sperm morphology. In the present study, both Mo and Cu were significant risk factors in the final model for sperm morphology (Table 6). The utility of μ statistics as a method to explore simultaneous metal relationships may thus be supported by the present findings. In the present study, however, we were able to demonstrate independent inverse relationships involving Mo and were also able to extend the analyses further to explore potential interactions between the two metals.

A limitation of the present study was the high percentage of blood samples with Mo concentrations < LOD, which hindered our ability to further investigate dose–response relationships and metal–metal interactions. Mo is quickly excreted in urine and has been found in higher concentrations in the kidneys of animals and humans compared with other organs (Vyskocil and Viau 1999). Thus, more sensitive biomarkers and assays, such as the measurement of Mo in urine, which has been able to quantify exposure in a high percentage of samples from the U.S. general population (CDC 2005), should be implemented in future studies of Mo exposure and adverse male reproductive outcomes.

The associations we found between other metals and semen quality were not as consistent throughout the various analytical approaches as those for Mo. We found suggestive evidence for nonmonotonic inverse associations between As and semen-quality parameters when metals were considered individually, but As was not retained in the models that considered multiple metals simultaneously. Our results for Pb were also inconsistent. We found a suggestive dose-dependent trend for increased odds of below-reference motility among increasing Pb quartiles that became weaker after adjustment for age and smoking. Pb was also retained as a predictor for increased odds for below-reference sperm concentration in models that considered multiple metals simultaneously (in addition to Mo and Mn). Telisman et al. (2007) recently reported nonoccupational Pb exposure, measured as blood Pb levels, to be associated with increased immature sperm and percentage of pathologic, wide, and round sperm in a study of Croatian men. Another study in Mexico found that Pb measured in spermatozoa or seminal fluid, but not in blood, was associated with decreased semen quality (Hernandez-Ochoa et al. 2005). However, median blood Pb levels in both studies (Croatia and Mexico) were higher than those in the present study, and perhaps a threshold exists for Pb-related effects on semen quality at Pb levels greater than those measured in our study of U.S. men.

A number of human studies have reported declines in semen quality associated with Cd in blood (Akinloye et al. 2006; Chia et al. 1992; Telisman et al. 2000) or semen (Dawson et al. 1998; Omu et al. 1995; Pant et al. 2003; Umeyama et al. 1986). However, in the present study we observed no associations between Cd concentrations in blood and semen quality. We found an inverse association between blood Cd and semen volume in crude regression models, which is consistent with previous reports among men in Singapore recruited through infertility clinics (Chia et al. 1992; Xu et al. 1993), but the relationship was no longer present after adjustment for smoking.

Another potential limitation of the present study was the collection of only a single blood and semen sample from each participant for measurement of metals and semen quality. However, for metals that are stored in the body, such as Pb, a single measure is likely a reliable marker of exposure over months or years (Egeghy et al. 2005). For metals that are rapidly excreted but enter the body primarily through diet (including drinking water and vitamin supplements), such as Mo, levels are also likely relatively stable over time because of consistent dietary patterns and drinking water sources (National Research Council 2006). The reliability of a single semen sample to represent a man’s semen quality over a longer period of time continues to be debated, although two recent reports have provided limited evidence that one sample may be adequately representative of semen quality over several weeks in large epidemiologic studies (Francavilla et al. 2007; Stokes-Riner et al. 2007).

As an indication of whether the concentrations of metals measured in blood in the present study were representative of those found among adult men in the wider general population, we compared the distribution of metal concentrations with those reported for adults in the Third National Report on Human Exposure to Environmental Chemicals (NRHEEC-III; CDC 2005). Of the metals measured in the present study, only Pb and Hg were measured in blood in the NRHEEC-III (several others, including Cd and Mo, were measured in urine). The distribution of blood Pb in the present study was similar to, although slightly lower than, those reported in NRHEEC-III. The median and 90th percentile values were 1.6 and 3.6 μg/dL among adults in the NRHEEC-III, compared with 1.5 and 3.2 μg/dL in the present study. Hg levels among adults from the NRHEEC-III were measured only in females, but the distribution of Hg values was similar to those in the present study of men. Recent population concentration distributions of blood Mo could not be located, although the maximum value in the present study (5.4 μg/L) was equal to the 70th percentile value reported in a 1968 U.S. study (Allaway et al. 1968). Results of that study suggested high regional variability, with a maximum value of 410 μg/L. More data on the sources, distribution, and time trends in Mo exposure are needed.

## Conclusion

Among a number of suggestive relationships between metals and semen quality that we observed in the present study, we found the most consistent evidence for an association between Mo concentrations in blood and reduced sperm concentration and morphology. These associations were robust to different statistical modeling strategies and the simultaneous consideration of other potentially harmful and/or beneficial metals. We also found qualitative evidence for interactions between Mo and low Cu or Zn on sperm concentration and morphology. Future work will involve examining these suggestive relationships with a larger sample size. Additional human epidemiologic studies, as well as mechanistic studies, are needed to confirm these findings.

## Figures and Tables

**Table 1:** Demographic categories by semen parameters^a^ (*n* = 219).

		Semen parameter		
Category	Comparison subjects (*n* = 73)^b^	Sperm concentration < 20 million/mL (*n* = 50)	Sperm < 50% motile (*n* = 74)	Sperm morphology < 4% normal (*n* = 97)
Age [years (mean ± SD)]	34.2 ± 5.6	34.6 ± 6.0	35.1 ± 4.8	33.9 ± 5.8
BMI (mean ± SD)	28.7 ± 5.0	30.0 ± 6.6	29.6 ± 7.1	30.6 ± 6.8
Race [no. (%)]				
White	59 (81)	37 (74)	53 (72)	68 (70)
Black	11 (15)	10 (20)	11 (15)	16 (16)
Other	3 (4)	3 (6)	9 (12)	12 (12)
Income [no. (%)]				
< $30,000	6 (8)	5 (11)	8 (11)	16 (17)
$30,000 to < $60,000	22 (31)	14 (30)	17 (24)	29 (32)
$60,000 to < $90,000	16 (22)	16 (34)	21 (30)	24 (24)
> $90,000	28 (39)	12 (26)	24 (34)	23 (25)
Smoking status [no. (%)]				
Never-smoker	43 (59)	28 (56)	35 (47)	55 (57)
Ever-smoker	30 (41)	22 (44)	39 (53)	42 (43)
Former	16 (22)	6 (12)	18 (24)	21 (22)
Current	14 (19)	16 (32)	21 (28)	21 (22)

a Information missing on race for two men and on income for eight men.

b Comparison group consists of men with all three semen parameters greater than the reference level.

**Table 2:** Distribution of metal concentrations in blood and semen-quality parameters (*n* = 219).

	Percentile						
Measure	10th	25th	50th	75th	90th	95th	Maximum
Metals							
As	2.83	5.80	8.10	10.0	11.5	12.4	25.5
Cd	< 0.2	< 0.2	0.20	0.40	0.90	1.30	2.80
Cr^a^	< 0.5	< 0.5	< 0.5	0.60	1.00	2.40	16.6
Cu	754	817	887	974	1,052	1,112	1,397
Pb (μg/dL)	0.80	1.10	1.50	2.00	3.20	4.20	16.2
Hg	0.30	0.60	1.10	2.30	3.90	5.40	14.5
Mo^b^	< 1.0	< 1.0	< 1.0	1.10	1.60	2.00	5.40
Tl	< 0.1	< 0.1	0.10	0.12	0.14	0.18	0.45
Zn	5,706	6,169	6,770	7,294	7,979	8,250	9,251
Semen parameters							
Semen volume (mL)	1.2	2.0	3.0	4.0	5.3	6.0	12.0
Sperm concentration (10^6^/mL)	7.9	21.0	42.7	77.0	115	147	324
Sperm motility (% motile)	34	45	55	63	71	75	93
Sperm morphology (% normal)^c^	0	1	3	6	9	11	16

Units are μg/L unless otherwise noted.

a For exposure groups used in models, 70th percentile = 0.6 μg/L; 85th percentile = 0.9 μg/L.

b For exposure groups used in models, 70th percentile = 1.0 μg/L; 85th percentile = 1.5 μg/L.

c Not measured in 31 men.

**Table 3:** Adjusted ORs (95% CIs) for below-reference semen parameters among metal exposure categories.

		Concentration		Motility		Morphology	
Metal percentile	No.^a^	No.^b^	OR (95% CI)	No.^b^	OR (95% CI)	No.^b^	OR (95% CI)
As							
< 25th	27	7	1	9	1	20	1
25th–50th	45	15	5.67 (1.74–18.4)	22	7.18 (2.43–21.2)	32	4.32 (1.72–10.9)
50th–75th	19	16	3.25 (1.11–9.53)	22	3.39 (1.27–9.07)	20	1.40 (0.59–3.31)
> 75th	17	12	2.78 (0.90–8.56)	21	3.80 (1.38–10.4)	25	1.97 (0.84–4.62)
*p*-Value for trend			0.13		0.04		0.39
Cd							
< 50th	39	25	1	34	1	50	1
50th–75th	18	14	1.05 (0.43–2.56)	18	1.06 (0.47–2.39)	20	0.87 (0.40–1.87)
> 75th	16	11	0.51 (0.15–1.74)	22	1.13 (0.41–3.14)	27	1.39 (0.54–3.59)
*p*-Value for trend			0.39		0.81		0.63
Cr							
< 70th	49	33	1	56	1	67	1
70th–85th	10	12	1.83 (0.70–4.78)	8	0.66 (0.24–1.82)	17	1.26 (0.53–3.00)
> 85th	14	5	0.52 (0.17–1.62)	10	0.68 (0.27–1.68)	13	0.68 (0.29–1.57)
*p*-Value for trend			0.22		0.32		0.51
Cu							
< 25th	25	9	1	10	1	19	1
25th–50th	13	17	3.83 (1.32–11.1)	27	5.27 (1.94–14.4)	20	2.07 (0.82–5.19)
50th–75th	19	9	1.31 (0.43–3.99)	16	2.30 (0.84–6.34)	29	2.04 (0.89–4.69)
> 75th	16	15	2.32 (0.79–6.80)	21	3.00 (1.10–8.19)	29	2.41 (1.10–5.84)
*p*-Value for trend			0.38		0.16		0.05
Pb							
< 25th	25	13	1	18	1	32	1
25th–50th	22	11	0.88 (0.32–2.44)	18	1.04 (0.43–2.53)	23	0.83 (0.37–1.87)
50th–75th	10	14	2.58 (0.86–7.73)	15	1.95 (0.70–5.46)	18	1.41 (0.54–3.67)
> 75th	16	12	1.16 (0.37–3.60)	23	1.66 (0.64–4.29)	24	1.18 (0.50–2.79)
*p*-Value for trend			0.38		0.18		0.51
Hg							
< 25th	16	19	1	26	1	34	1
25th–50th	17	10	0.52 (0.18–1.49)	12	0.44 (0.16–1.16)	17	0.48 (0.19–1.17)
50th–75th	17	10	0.47 (0.16–1.36)	21	0.74 (0.30–1.83)	25	0.69 (0.29–1.64)
> 75th	23	11	0.36 (0.12–1.03)	15	0.35 (0.14–0.90)	21	0.43 (0.18–1.02)
*p*-Value for trend			0.06		0.07		0.1
Mo							
< 70th	54	31	1	53	1	66	1
70th–85th	13	9	1.39 (0.52–3.71)	9	0.80 (0.29–1.96)	12	0.78 (0.32–1.89)
> 85th	6	10	3.48 (1.12–10.8)	12	2.24 (0.77–6.49)	19	2.61 (0.97–7.03)
*p*-Value for trend			0.04		0.27		0.12
Tl							
< 50th	41	21	1	32	1	46	1
50th–75th	17	18	2.01 (0.84–4.80)	23	1.75 (0.80–3.85)	27	1.40 (0.67–2.93)
> 75th	15	11	1.45 (0.56–3.80)	19	1.51 (0.65–3.50)	24	1.41 (0.65–3.05)
*p*-Value for trend			0.27		0.23		0.33
Zn							
< 25th	20	10	1	20	1	24	1
25th–50th	17	15	1.72 (0.60–4.89)	16	0.92 (0.36–2.35)	25	1.23 (0.52–2.89)
50th–75th	18	12	1.46 (0.49–4.30)	17	1.03 (0.41–2.58)	25	1.18 (0.50–2.80)
> 75th	18	13	1.62 (0.56–4.68)	21	1.30 (0.53–3.19)	23	1.07 (0.45–2.52)
*p*-Value for trend			0.47		0.55		0.91

ORs were adjusted for age and current smoking comparison group was men with all three semen parameters greater than reference values.

a Number of men with value greater than reference for all three semen parameters.

b Number of men with value less than reference for single semen parameter.

**Table 4:** Adjusted regression coefficients (95% CIs) for change in semen-quality parameters associated with metal categories.

	Semen parameter			
Metal percentile	Volume^a^	Concentration^b^	Motility	Morphology
As				
< 25th	0	0	0	0
25th–50th	0.24 (−0.33 to 0.81)	−0.39 (−0.79 to 0.02)	−3.50 (−9.07 to 2.06)	−2.54 (−3.91 to −1.17)
50th–75th	0.15 (−0.42 to 0.73)	−0.35 (−0.75 to 0.06)	−4.87 (−10.4 to 0.69)	−0.59 (−1.98 to 0.80)
> 75th	0.28 (−0.30 to 0.85)	−0.24 (−0.65 to 0.17)	−3.91 (−9.51 to 1.69)	−1.08 (−1.98 to 0.80)
*p*-Value for trend	0.42	0.29	0.14	0.52
Cd				
< 50th	0	0	0	0
50th–75th	−0.32 (−0.82 to 0.19)	0.01 (−0.35 to 0.37)	−1.07 (−6.01 to 3.86)	0.45 (−0.86 to 1.76)
> 75th	−0.16 (−0.78 to 0.46)	0.30 (−0.15 to 0.74)	1.64 (−4.50 to 7.79)	−0.53 (−2.07 to 1.00)
*p*-Value for trend	0.41	0.26	0.76	0.68
Cr				
< 70th	0	0	0	0
70th–85th	0.06 (−0.51 to 0.64)	−0.44 (−0.84 to −0.04)	0.08 (−5.45 to 5.60)	−1.25 (−2.72 to 0.21)
> 85th	0.09 (−0.48 to 0.67)	0.20 (−0.20 to 0.60)	1.75 (−3.82 to 7.33)	−0.03 (−1.45 to 1.38)
*p*-Value for trend	0.32	0.86	0.57	0.58
Cu				
< 25th	0	0	0	0
25th–50th	−0.26 (−0.83 to 0.31)	−0.22 (−0.62 to 0.18)	−5.31 (−10.9 to 0.26)	−0.76 (−2.22 to 0.70)
50th–75th	−0.39 (−0.96 to 0.18)	0.16 (−0.25 to 0.56)	−2.82 (−8.41 to 2.78)	−1.52 (−2.94 to −0.10)
> 75th	−0.61 (−1.19 to −0.03)	−0.34 (−0.75 to 0.07)	−1.04 (−6.72 to 4.64)	−1.98 (3.43 to −0.54)
*p*-Value for trend	0.04	0.36	0.93	0.004
Pb				
< 25th	0	0	0	0
25th–50th	−0.43 (−0.99 to 0.13)	−0.15 (−0.55 to 0.25)	0.96 (−4.52 to 6.45)	0.71 (−0.69 to 2.11)
50th–75th	−0.28 (−0.87 to 0.31)	−0.25 (−0.67 to 0.18)	2.00 (−3.87 to 7.88)	−0.83 (−2.34 to 0.67)
> 75th	0.17 (−0.41 to 0.74)	0.02 (−0.39 to 0.43)	1.10 (−4.56 to 6.75)	−0.16 (−1.58 to 1.26)
*p*-Value for trend	0.92	0.97	0.64	0.42
Hg				
< 25th	0	0	0	0
25th–50th	−0.07 (−0.65 to 0.50)	0.05 (−0.36 to 0.46)	0.40 (−5.22 to 6.02)	0.33 (−1.14 to 1.80)
50th–75th	−0.06 (−0.61 to 0.49)	0.16 (−0.23 to 0.56)	−2.88 (−8.26 to 2.50)	−0.18 (−1.52 to 1.17)
> 75th	0.20 (−0.37 to 0.76)	0.04 (−0.36 to 0.45)	1.71 (−3.75 to 7.17)	0.45 (−0.97 to 1.88)
*p*-Value for trend	0.88	0.68	0.86	0.71
Mo				
< 70th	0	0	0	0
70th–85th	0.10 (−0.47 to 0.68)	−0.15 (−0.55 to 0.26)	2.83 (−2.78 to 8.43)	−0.24 (−1.75 to 1.26)
> 85th	−0.04 (−0.64 to 0.55)	−0.29 (−0.72 to 0.13)	−5.39 (−11.2 to 0.39)	−1.39 (−2.85 to 0.07)
*p*-Value for trend	0.99	0.15	0.2	0.07
Tl				
< 50th	0	0	0	0
50th–75th	−0.12 (−0.61 to 0.37)	−0.10 (−0.46 to 0.25)	−2.26 (−7.09 to 2.56)	−0.12 (−1.36 to 1.12)
> 75th	−0.16 (−0.67 to 0.34)	0.01 (−0.35 to 0.36)	−1.20 (−6.10 to 3.71)	−0.75 (−2.01 to 0.52)
*p*-Value for trend	0.5	0.93	0.53	0.27
Zn				
< 25th	0	0	0	0
25th–50th	−0.21 (−0.77 to 0.36)	−0.20 (−0.60 to 0.21)	2.32 (−3.24 to 7.88)	0.17 (−1.25 to 1.59)
50th–75th	−0.13 (−0.70 to 0.45)	−0.39 (−0.80 to 0.01)	4.26 (−1.37 to 9.90)	0.09 (−1.34 to 1.53)
> 75th	−0.54 (−1.12 to 0.03)	−0.14 (−0.54 to 0.27)	0.29 (−5.32 to 5.89)	0.34 (−1.10 to 0.07)
*p*-Value for trend	0.09	0.36	0.77	0.68

Regression coefficients were adjusted for age and current smoking.

a One extremely high semen volume (12 mL) was excluded.

b Sperm concentration was natural-log transformed.

**Table 5:** Final logistic regression models^a^ for below-reference sperm concentration and morphology when considering multiple metals and other covariates.

Variable	OR (95% CI)	*p*-Value
Sperm concentration		
Mo		
70th–85th percentile	2.23 (0.66–7.60)	
> 85th percentile	6.26 (1.57–25.0)	0.07*
Pb		
25th–50th	0.89 (1.57–2.89)	
50th–75th	3.94 (1.15–13.6)	
> 75th	2.48 (0.59–10.4)	0.07*
Mn		
25th–50th	0.70 (0.22–2.21)	
50th–75th	1.39 (0.32–6.12)	
> 75th	2.90 (0.83–10.2)	0.03*
Cd		
50th–75th	0.77 (0.26–0.75)	
> 75th	0.14 (0.03–0.75)	0.02*
Hg		
25th–50th	0.53 (0.16–1.71)	
50th–75th	0.38 (0.11–1.29)	
> 75th	0.24 (0.08–0.75)	0.02*
Smoking status^b^		
Current smoker	7.11 (1.98–25.6)	0.01
Sperm morphology		
Mo		
70th–85th percentile	0.91 (0.37–2.24)	
> 85th percentile	3.44 (1.23–9.67)	0.04*
BMI		
Unit increase	1.06 (1.00–1.12)	0.02
Household income (US$)^c^		
30,000–60,000	0.46 (0.16–1.53)	
60,000–90,000	0.51 (0.17–1.53)	
> 90,000	0.28 (0.10–0.80)	0.07*

a Backward elimination; α = 0.1.

b Reference group, current nonsmokers.

c Reference group, household income < $30,000/year.

* *p*-Value for trend.

**Table 6:** Final semen volume and sperm morphology linear regression models,^a^ considering multiple metals and other covariates.

Variable	Regression coefficient (95% CI)	*p*-Value
Semen volume		
As		
25th–50th percentile	0.43 (−0.13 to 0.99)	
50th–75th percentile	0.39 (−0.19 to 0.97)	
> 75th percentile	0.54 (−0.07 to 1.15)	0.05*
Zn		
25th–50th	−0.36 (−0.91 to 0.20)	
50th–75th	−0.24 (−0.82 to 0.34)	
> 75th	−0.78 (−1.38 to −0.18)	0.02*
Smoking status		
Current smoker	−0.42 (−0.91 to 0.07)	0.09
Household income^b^		
$30,000–$60,000	0.40 (−0.24 to 1.04)	
$60,000–$90,000	0.82 (0.15 to 1.49)	
> $90,000	0.83 (0.18 to 1.49)	0.07*
Race^c^		
White	0.49 (0.01 to 0.98)	0.04
Sperm morphology		
Mo		
70th–85th	−0.26 (−1.74 to 1.22)	
> 85th	−1.59 (−3.03 to −0.15)	0.03*
Cu		
25th–50th	−0.84 (−2.28 to 0.60)	
50th–75th	−1.45 (−2.88 to −0.01)	
> 75th	−1.50 (−2.98 to −0.02)	0.05*
BMI		
Unit increase	−0.05 (−0.14 to 0.03)	0.08

a Backward elimination; same results obtained with forward selection and stepwise procedures.

b Reference group, household income < $30,000/year.

c Reference group, nonwhite.

* *p*-Value for trend.
